# Systemic and Cerebral Iron Homeostasis in Ferritin Knock-Out Mice

**DOI:** 10.1371/journal.pone.0117435

**Published:** 2015-01-28

**Authors:** Wei Li, Holly J. Garringer, Charles B. Goodwin, Briana Richine, Anthony Acton, Natalia VanDuyn, Barry B. Muhoberac, Jose Irimia-Dominguez, Rebecca J. Chan, Munro Peacock, Richard Nass, Bernardino Ghetti, Ruben Vidal

**Affiliations:** 1 Department of Pathology and Laboratory Medicine, Indiana University School of Medicine, Indianapolis, Indiana, 46202, United States of America; 2 Department of Pediatrics, Indiana University School of Medicine, Indianapolis, Indiana, 46202, United States of America; 3 Department of Medicine, Indiana University School of Medicine, Indianapolis, Indiana, 46202, United States of America; 4 Department of Pharmacology and Toxicology, Indiana University School of Medicine, Indianapolis, Indiana, 46202, United States of America; 5 Department of Chemistry and Chemical Biology, Indiana University-Purdue University Indianapolis, Indianapolis, Indiana, 46202, United States of America; Lady Davis Institute for Medical Research/McGill University, CANADA

## Abstract

Ferritin, a 24-mer heteropolymer of heavy (H) and light (L) subunits, is the main cellular iron storage protein and plays a pivotal role in iron homeostasis by modulating free iron levels thus reducing radical-mediated damage. The H subunit has ferroxidase activity (converting Fe(II) to Fe(III)), while the L subunit promotes iron nucleation and increases ferritin stability. Previous studies on the H gene (*Fth*) in mice have shown that complete inactivation of *Fth* is lethal during embryonic development, without ability to compensate by the L subunit. In humans, homozygous loss of the L gene (*FTL*) is associated with generalized seizure and atypical restless leg syndrome, while mutations in *FTL* cause a form of neurodegeneration with brain iron accumulation. Here we generated mice with genetic ablation of the *Fth* and *Ftl* genes. As previously reported, homozygous loss of the *Fth* allele on a wild-type *Ftl* background was embryonic lethal, whereas knock-out of the *Ftl* allele (*Ftl^-/-^*) led to a significant decrease in the percentage of *Ftl^-/-^* newborn mice. Analysis of *Ftl^-/-^* mice revealed systemic and brain iron dyshomeostasis, without any noticeable signs of neurodegeneration. Our findings indicate that expression of the H subunit can rescue the loss of the L subunit and that H ferritin homopolymers have the capacity to sequester iron *in vivo*. We also observed that a single allele expressing the H subunit is not sufficient for survival when both alleles encoding the L subunit are absent, suggesting the need of some degree of complementation between the subunits as well as a dosage effect.

## Introduction

Ferritin, an iron storage protein, is composed of 24 subunits that self-assemble into a 480 kDa hollow sphere of ∼110 Å outer and ∼80 Å inner diameter, which can store up to 4500 atoms of iron as a ferrihydrite biomineral [1, 2]. Human ferritin is usually heteropolymeric with a variable ratio of ferritin light polypeptides (FTL or L) and heavy polypeptides (FTH1 or H). The H and L subunits are conformationally equivalent with 54% sequence identity and slightly different masses. The H subunit contains the ferroxidase center, which oxidases Fe(II) to Fe(III). The L subunit does not have catalytic activity, but offers acidic residues on the cavity surface that facilitate iron nucleation enhancing biomineral formation [2]. Each cell type fine tunes the ratio of H to L subunits for optimal physiological function. The ferroxidase activity is essential for iron incorporation into ferritin, contributing to the maintenance of the redox status of the cells by removing Fe(II). Improperly coordinated Fe(II) has the potential to convert hydrogen peroxide and superoxide into the highly toxic hydroxyl radical, which can attack proteins, lipids, and DNA causing oxidation, fragmentation and crosslinking leading to their loss of function [2].

The 5’-untranslated regions flanking both ferritin genes are unusually long and contain nearly identical stem-loop structures known as iron responsive elements (IREs).

Binding of the cytoplasmic RNA-binding protein aconitase 1 (ACO1), also known as iron regulatory protein 1 (IRP1), and the iron-responsive element-binding protein

2 (IREB2 or IRP2) to the IREs regulates the translation of ferritin mRNAs [3]. Both IRP1 and IRP2 are ubiquitously expressed, with the iron concentration and oxidative status of the cell determining the ability of the IRPs to bind to an IRE [3]. Mutations in the IRE sequence of the ferritin genes cause disease by modifying the efficiency of mRNA translation [3]. A mutation in the IRE sequence of the *FTH1* gene in a Japanese family leads to the development of an autosomal dominant condition (hemochromatosis type 5) presenting with decreased levels of H polypeptides and iron overload [4], while mutations in the IRE sequence of the *FTL* gene have been found associated with the development of hereditary hyperferritinemia cataract syndrome, a disorder characterized by high levels of serum ferritin and early onset bilateral cataract, but no alterations of iron metabolism [5, 6].

Mutations in the coding sequence of the *FTH1* gene itself have not so far been reported, but mutations in the coding sequence of the *FTL* gene have been reported in the autosomal dominant disorder neuroferritinopathy or hereditary ferritinopathy (HF) [2]. HF has a clinical phenotype characterized by a progressive movement disorder, behavioral disturbances, and cognitive impairment. The main pathologic findings in this condition are cystic cavitation of the basal ganglia, the presence of ferritin inclusion bodies (IBs) in glial cells and neurons in the central nervous system (CNS), and substantial iron deposition. All mutations found in patients with HF occur in exon 4 of the *FTL* gene, leading to the generation of an L subunit with a longer than normal C-terminal sequence [1, 2]. The incorporation of the mutant subunit in ferritin causes a loss of normal ferritin function by decreasing iron incorporation (triggering intracellular iron accumulation and overproduction of ferritin polypeptides), and a gain of a toxic function through radical production, ferritin aggregation, and oxidative stress [1, 2, 7–9]. Although HF is relatively rare, its study is particularly important since in HF there is a direct genetic link between abnormal iron metabolism and neurodegeneration [1, 2, 10, 11]. More recently, the complete loss of functional L subunits was reported in an individual homozygous for a nonsense mutation at codon 104 (*p*.*Glu104Ter*) of the *FTL* gene. The patient had seizures during infancy and presented with an atypical form of restless leg syndrome (RLS), with mild neuropsychological impairment and a reduced intelligence quotient. In this patient, serum ferritin was undetectable; however, normal values were observed for hemoglobin concentration, mean corpuscular volume (MCV), mean corpuscular hemoglobin concentration (MCHC), total red blood cells (RBC), and haptoglobin. Serum iron levels, transferrin, and transferrin-saturation were also within the normal range. Normal iron stores in the liver were observed by T2^*^ MRI, whereas brain MRI did not show any iron deposition in the basal ganglia. Protein studies suggest that the truncated peptide is unable to assemble into ferritin polymers [12].

Herein, we analyzed iron metabolism in mice in which the ferritin genes were disrupted by homologous recombination to provide further understanding of the role(s) that the ferritin subunits play in iron homeostasis, with particular focus on the L subunit.

## Material and Methods

### Ethics Statement

This study was carried out in strict accordance with the Guidelines for the Care and Use of Laboratory Animals of the National Institutes of Health. The protocol was approved by the Indiana University School of Medicine Institutional Animal Care and Use Committee (Protocol Number: 10149). All surgeries were performed under anesthesia, and all efforts were made to minimize animal suffering. Mice were anesthetized with acepromazine (2–5 mg/kg) + ketamine (100 mg/kg) given intraperitoneally. The animals remained anesthetized during the entire procedure and were euthanized without awakening.

### Gene targeting and generation of mutant mice

**Fig 1:**
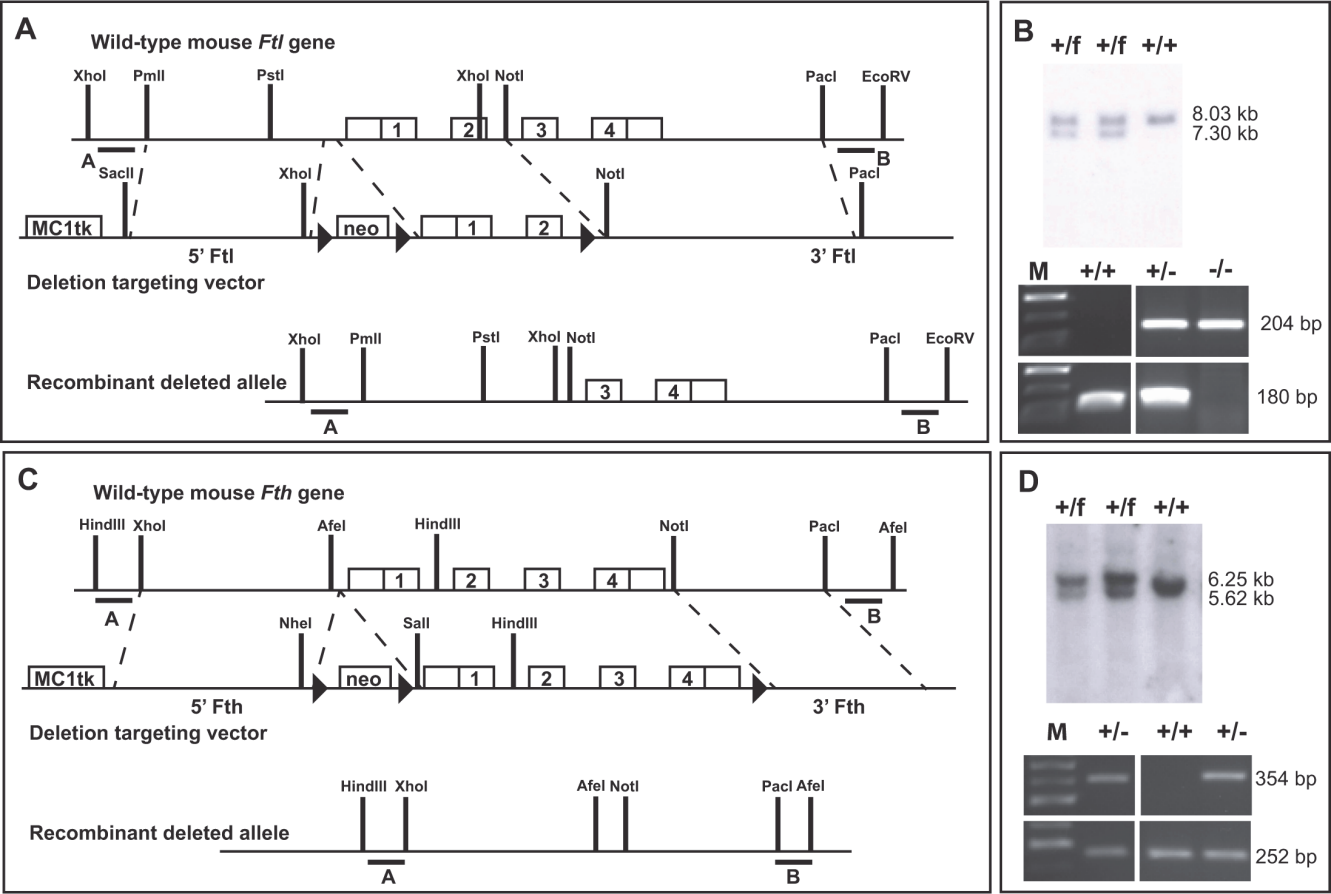
Strategy employed for the inactivation of the murine ferritin light and heavy genes. For the inactivation of the *Ftl* gene, exons 1 and 2 were deleted (A). The deletion targeting vector contained the *MC1tk* (thymidine kinase) expression cassette, 5.72 kb of 5’ *Ftl* homology, a *pGK-neo* (neomycin-resistance gene) expression cassette, 0.83 kb of the *Ftl* gene containing exons 1 and 2, and 5.74 kb of 3’ *Ftl* homology. Three loxP sites were introduced by targeted mutagenesis between the *neo* cassette and after exon 2 of *Ftl*. Homologous recombination in ES cells identified by Southern blot hybridization analysis using the external probe A after digestion with XhoI. The wild-type allele has 8.03 kb and the floxed allele has 7.30 kb (B). Germline chimeras were crossed with C57Bl/6 females, and mice carrying the floxed *Ftl* (*Ftl* ^*f/+*^) allele were then crossed with mice expressing the *Cre* recombinase. Mice showing the deletion of exons 1 and 2 of *Ftl* and the *neo* cassette were crossed with C57Bl/6 mice to remove the *Cre* allele. Mice were maintained in C57Bl/6 background and the presence of the *Ftl* allele determined by PCR, resulting in a 180-bp fragment for the wild-type allele and in a 204-bp fragment for the knock-out allele (B). The four exons of the mouse *Fth* gene were targeted for deletion (C). The deletion targeting vector contained the *MC1tk* expression cassette, 4.44 kb of 5’ *Fth* homology, a *pGK-neo* expression cassette, 2.4 kb of the *Fth* gene containing all four exons, and 5.0 kb of 3’ *Fth* homology. Three loxP sites were introduced by targeted mutagenesis between the neo cassette and after exon 4 of *Fth*. Homologous recombination in ES cells identified by Southern blot hybridization analysis using the external probe A after digestion with HindIII. The wild-type allele has 6.25 kb and the floxed *Fth* (*Fth* ^*f/+*^) allele has 5.62 kb (D). Germline chimeras were crossed with C57Bl/6 females, and mice carrying the floxed *Fth* allele were then crossed with mice expressing the *Cre* recombinase. Mice showing complete loss of *Fth* and the *neo* cassette were crossed with C57Bl/6 mice to remove the *Cre* allele. Mice were maintained in C57Bl/6 background and the presence of the *Fth* allele determined by PCR, resulting in a 252-bp fragment for the wild-type allele and in a 354-bp fragment for the knock-out allele (D). The symbol ► represents the loxP sites; f, floxed alleles.

The structure of the gene-targeting vectors is shown in Fig. 1. Two targeting vectors were constructed to delete the murine wild-type *Ftl and Fth* genes by homologous recombination. The *Ftl* gene targeting vector contained a 5.7 kb *Ftl* 5’ flanking region that was derived from a murine genomic *Ftl* subclone (RPCI23.C 303G13, Invitrogen) corresponding to mouse chromosome 7. The 5’ flank extended from a *Sac*II site to an *Xho*I site in the 5’ region of the *Ftl* gene, followed by a *neomycin resistance (neo* ^*r*^ *)* cassette (1.6 kb).The *Ftl* 3’ flank extended from a *Not*I site located in intron 2 of the *Ftl* gene to a *Pac*I site 5.7 kb further downstream. LoxP sequences were inserted into the 5’-untranslated region and the 3’-end of the neomycin gene-expressing cassette and at the end of exon 2 of the *Ftl* gene (Fig. 1A). The targeting vector for the *Fth* gene contained a 4.4 kb *Fth* 5’ flanking region that was derived from a murine genomic *Fth* subclone (RPCI23.C 64I17, Invitrogen) corresponding to mouse chromosome 19. The 5’ flank extended from an *Xho*I site to a *Nhe*I site in the 5’ region of the *Fth* gene, followed by the *neo* ^*r*^ cassette. The *Fth* 3’ flank extended from a *Sal*I site located in the 5’ of the *Fth* gene and included the entire *Fth* gene (2.4 kb). The 3’ flank ended on a *Pac*I site 5.0 kb downstream of the *Fth* gene. LoxP sequences were inserted into the 5’-untranslated region and the 3’-end of the neomycin gene-expressing cassette and at the end of exon 4 of the *Fth* gene (Fig. 1B). Both targeting vectors were linearized with *Pme*I and electroporated into 129/SvJ embryonic stem cells (ES). Targeted clones were obtained by positive/negative selection in the presence of G418 (Geneticin, GIBCO-BRL) at the Indiana University Transgenic and Knock-out Mouse Core Facility. ES cells were injected into C57BL/6 blastocysts and chimeric males were bred to C57BL/6 females. Heterozygous targeted mice (*Ftl* ^*+/neo*^ and *Fth1* ^*+/neo*^) were immediately bred to a *Cre splicer* mouse (EIIa-*Cre* mice) to remove the neomycin resistance cassette and ferritin coding sequences, resulting in *Ftl* ^+/-^ and *Fth* ^+/-^ mice. Correctly targeted mice were identified by Southern blot hybridization of genomic DNA digested with *Xho*I for the *Ftl* allele and *Hind*III for the *Fth* allele using DNA probes external to the ferritin sequences contained in the targeting vector, and by PCR analysis of tail tip genomic DNA. Animals were backcrossed to C57Bl/6 mice to remove the *Cre* allele and maintained in C57Bl/6 background. Mice were fed a normal iron diet.

### Polymerase Chain Reaction (PCR) Genotyping of mice

Mice were genotyped for the presence of the neomycin resistance cassette using a *neo*-specific primer set (forward primer: 5’-AAG CGG CCA TTT TCC ACC AT-3’; reverse primer: 5’-TGC CGC GCT GTT CTC CTC T-3’). After PCR amplification, the presence of a *neo*-specific product in *Ftl* ^*+/neo*^ and *Fth1* ^*+/neo*^ samples was determined after separation of the reaction mixture on a 1% agarose gel. Mice were also genotyped for *Cre* by PCR. The PCR reaction contained a *Cre*-specific primer set (forward primer: 5’-TGA CGT AGT TTT CGC GCT TAA-3’; reverse primer: 5’-GAA CCT CAT CAC TCG TTG CA-3’). After PCR amplification, the presence of *Cre*-specific product in mice carrying the *Cre* allele was determined after separation of the reaction mixture on a 1% agarose gel. The presence of the ferritin alleles was also confirmed by PCR amplification. For the *Ftl* mice, the forward primer: 5’-CCT CAG CTC CGG ATT GGT-3’ and the reverse primer: 5’-GTT CCG TTC AAG CAC TGT TG-3’ were used to detect the wild-type allele (180 bp). The forward primer and the reverse primer: 5’-ACT AGT CCT GCC ACC ACT CC-3’ were used to detect the knock-out allele (204 bp). Genotyping was also performed by PCR on 11.5 and 13.5 post coitus *Ftl* ^*-/-*^ embryos. Embryos were removed from the uterus of pregnant females, washed with water, and then transferred into Eppendorf cups containing 10 ml of water and 7 ml of phosphate buffered saline (PBS). Cell DNA was released by successive dry ice freezing and boiling steps followed by a 30-min incubation at 56°C in the presence of 3 ml of proteinase K (10 mg/ml). A final incubation was done at 95°C for 10 min, and samples were kept at-80°C. The whole lysate was used for PCR. For the *Fth* mice, the forward primer: 5’-TCT TGC AGA AGC TCA GAG CC-3’ and the reverse primer: 5’-GTT CCG TTC AAG CAC TGT TG-3’ were used to detect the wild-type allele (252 bp). The forward primer and the reverse primer: 5’-CCA AGA GTA CTT AAT AGT CCT GCC TG-3’ were used to detect the knock-out allele (354 bp).

### Serum Biochemistry and Hematological Analyses

Blood samples were obtained prior to perfusions by cardiac puncture. Serum was separated by centrifugation and used to determine Transferrin (Tf) saturation and iron using a COBAS MIRA Plus Chemistry Analyzer (Roche Diagnostics). A complete blood cell count (CBC) was performed on whole blood using a Mascot HemaVet950FS automated processor.

### Histology and immunohistochemistry

After anesthesia, mice were transcardially perfused with 0.9% saline and brains were fixed by immersion in 4% paraformaldehyde solution for 24 h at 4°C. The brains were embedded in paraffin and sectioned.

Eight-micrometer-thick sections were stained by the Hematoxylin-Eosin (H&E) method. In addition, Perls’ method for ferric iron was used as described [13]. Immunohistochemical labeling was also carried out following published protocols [13]. For immunohistochemistry, sections were incubated overnight at 4°C with the primary antibodies in blocking solution. Immunostaining was visualized using the avidin-biotin system (Vectastain;Vector Laboratories, Burlingame, CA, USA) and 3,3’-diaminobenzidine (Sigma) as the chromogen. The sections were counterstained with cresyl violet or H&E, and images were captured by a digital camera coupled to a Leica DM4000B microscope (Leica Microsystems, Germany).

### Western blot analysis

Cytoplasmic fractions were prepared from brain cortex and liver from 7-month-old male and female mice using the CelLytic NuCLEAR Extraction Kit (Sigma) following the manufacturer’s procedures. Protein extracts were aliquoted and stored at-80°C until used. Protein concentration was determined by using the Pierce BCA Protein Assay kit (Thermo Scientific). Between 50 to 150 μg of protein was run in denaturing 10% Bis-Tris SDS-polyacrylamide gels (NuPAGE Novex, Life Technologies) and transferred to Immobilon-P membranes (GE Healthcare, Piscataway, NJ). Membranes were blocked for 1 h in 5% low fat dried milk in TBS containing 0.1% Tween-20 (TBS-T) and then incubated for 1 h with the primary antibody. After washing in TBS-T, the membranes were incubated with peroxidase-conjugated secondary antibody (GE Healthcare) (1:5,000) for 1 h. Membranes were developed using the ECL chemiluminescent detection system (GE Healthcare). Equal protein load was confirmed after reprobing the membrane using anti-β-actin antibodies. The films were scanned and the densities of the bands measured using NIH ImageJ Software. The densities of the bands were normalized against those of β-actin and the mean ratios calculated. Statistical analysis was performed using GraphPad Prism (GraphPad Software, San Diego, CA).

### Antibodies

The following antibodies were used: anti-Glial Fibrillary Acidic Protein (GFAP) (ASTRO6, Thermo Scientific), anti-ionized calcium binding adapter molecule 1 (Iba1) (Wako Chemicals), anti-L antibody (ab109373; Abcam, Cambridge, MA), anti-H antibody (ab65080; Abcam, Cambridge, MA), and anti-β actin (1:10000; Sigma, St. Louis, MO).

### RNA isolation and multiplex expression analysis

Mice were anesthetized, transcardially perfused with 0.9% saline, and the brain and a liver sample removed. The cerebral cortex (CTX) was microdissected. CTX and liver samples were placed in 500ul of RNA later (Qiagen) and frozen at-20°C. RNA was isolated from the CTX using RNeasy Lipid Tissue Mini Kit (Qiagen) and from the liver using the RNeasy Mini Kit (Qiagen) according to the manufacturer protocol. Samples were treated on column with the RNase free DNase Kit (Qiagen) according to the manufacturer instructions. Reverse transcription was performed on 50 ng of total RNA for each sample followed by multiplex PCR, and fragment separation by capillary electrophoresis using the GeXP Chemistry Protocol (Beckman Coulter, Fullerton, CA). Gene specific primer pairs (without universal tags) used in RT-PCR are listed in S1 Table. Fragments were separated using a CEQ 8000 Automated Capillary DNA sequencer/Genetic Analysis Systems (Beckman Coulter), and analyzed using the GenomeLab GeXP Genetic Analysis System (Beckman Coulter) using the following fragment analysis parameters: slope threshold = 0.9999, peak height threshold = 800 rfu, peak size < 375, peak size > 150, dye = D4. Multiplex-specific fragments were selected by applying exclusion filters and the data exported to eXpress Analysis software, where they were normalized against the mouse *polymerase II polypeptide A* (*Polr2a*) gene as described [14]. Relative mRNA level values for each of the triplicates for each sample were averaged and the mean for the replicates were compared between knock-out and control mice by an unpaired two-tailed t-test using GraphPad Prism. Differences in relative mRNA levels with p-values < 0.05 were considered statistically significant. Data are reported as mean ± standard deviation (SD).

### Measurement of Total Iron

CTX, striatum, and liver were dissected as previously reported [13–15] and the wet weight of each sample was obtained. Tissue was transferred to a Teflon digestion vessel and 2 ml of 50% HNO_3_ was added. Samples were digested in the MARS Xpress system (CEM) for 15 min at 200°C and then diluted with H_2_O to bring the final acid concentration to 10% as described [16]. For introduction into the instrument, samples were diluted to 12.5–200 μl of digested tissue sample per ml with 2% HNO_3_. Total iron content of each sample was determined by analysis with the X Series ICP-MS (Thermo Fisher Scientific) in Collision Cell Technology (CCT) mode using H/He as the gas. A standard curve for Fe was prepared from 0–100 ppb and internal standards Ga (50 ppb) and Y (100 ppb) were used. The iron content was normalized to wet weight and the results were reported as nmol Fe/g tissue (wet weight). Statistical analysis was done using GraphPad Prism.

## Results

### Generation and viability of mice with null ferritin alleles

We generated mice in which the murine ferritin genes were disrupted by homologous recombination. In addition, our targeting strategy entailed the generation of “floxed” ferritin alleles for conditional knock-out experiments (Fig. 1A, C). Ferritin knock-out mice were generated by the removal of exons 1 and 2 of the murine *Ftl* gene, exons 1 through 4 of the murine *Fth* gene, and the neomycin resistance gene by *Cre*-recombination using EIIA-*Cre* mice. Mice carrying the knock-out alleles were identified by Southern blot hybridization of genomic DNA digested with *Xho*I for the *Ftl* allele and *Hind*III for the *Fth* allele using DNA probes external to the ferritin sequences contained in the targeting vector, and by PCR analysis of tail tip genomic DNA (Fig. 1B, D). Mice were mated with C57Bl/6 mice to remove the *Cre* recombinase allele. Heterozygous knock-out mice were further backcrossed and maintained on a C57Bl/6 background. PCR was used to determine the presence of the *Cre* allele and the genotype of the mice. Heterozygous mice (*Fth* ^+/-^ and *Ftl* ^+/-^) presented with no growth abnormalities, were fertile and thrived at appropriate age, as did their wild-type littermates. No susceptibility to infection was noted. Mice had good body condition with adequate body fat and no discharges or secretions from nostrils, conjunctiva, aural, urogenital or anal openings. The animals were active and alert. As previously reported [17, 18], crossing of *Fth* ^+/-^ heterozygous mice did not lead to the generation of homozygous knock-out mice. We genotyped over 200 new born mice, with ratios of 36% wild-type (*Fth* ^+/+^), 64% *Fth* ^+/-^ heterozygous and 0% *Fth* ^-/-^ homozygous mice. Different from *Fth* ^+/-^ heterozygous mice, crossing of *Ftl* ^+/-^ heterozygous mice led to the production of *Ftl* ^-/-^ homozygous mice, but at an abnormal Mendelian frequency. Genotyping of new born mice showed a frequency of 26% wild-type (*Ftl* ^+/+^), 57% *Ftl* ^+/-^ heterozygous and 17% *Ftl* ^-/-^ homozygous mice, which was significantly different from the expected 1:2:1 ratio. Moreover, genotyping of new born mice generated by mating of *Ftl* ^+/-^ heterozygous and *Ftl* ^-/-^ homozygous mice showed a frequency of 74% *Ftl* ^+/-^ heterozygous and 26% *Ftl* ^-/-^ homozygous mice, also different from the expected 1:1 ratio. Genotyping of embryos at embryonic days 11.5 and 13.5 showed a ∼50% reduction of *Ftl* ^-/-^ homozygous embryos. The remaining *Ftl* ^-/-^ embryos were phenotypically normal and survived until birth. Mice developed normally; however, at approximately 2 months of age, ∼50% of homozygous *Ftl* knock-out mice exhibit a head tilt and a circling behavior in the same direction, which persisted throughout the life of the animal. The circling behavior become more prominent and present in more mice as animals aged (observed in approximately 80% of adult *Ftl* ^-/-^ mice). This phenotype was not observed in wild-type (*Ftl* ^+/+^) littermates. Crossing of *Fth* ^+/-^ heterozygous mice and *Ftl* ^+/-^ heterozygous mice led to the generation of double *Fth* ^+/-^/*Ftl* ^+/-^ heterozygous mice. These mice were indistinguishable from control littermates. Mating of *Fth* ^+/-^/*Ftl* ^+/-^ double heterozygous mice led to the production of *Fth* ^+/-^/*Ftl* ^+/-^ heterozygous mice (25%), *Fth* ^+/-^/*Ftl* ^+/+^ mice (27%), *Fth* ^+/+^/*Ftl* ^+/-^ mice (14%), *Fth* ^+/+^/*Ftl* ^+/+^ mice (27%), and *Fth* ^+/+^/*Ftl* ^-/-^ mice (7%). After several rounds of matings, we failed to obtain *Fth* ^-/-^/*Ftl* ^-/-^ double homozygous mice, *Fth* ^-/-^/*Ftl* ^+/+^ mice, *Fth* ^-/-^/*Ftl* ^+/-^ mice, and *Fth* ^+/-^/*Ftl* ^-/-^ mice. *Fth* ^+/-^/*Ftl* ^+/-^ double heterozygous mice were also crossed with *Ftl* knockout mice (*Fth* ^+/+^/*Ftl* ^*-*/-^ mice). After several rounds of matings using multiple breeders, we observed the production of mice with an abnormal Mendelian frequency from the expected 1:1:1:1 ratio for the four possible genotypes. We obtained *Fth* ^+/-^/*Ftl* ^+/-^ mice (40%), *Fth* ^+/+^/*Ftl* ^-/-^ mice (31%), and *Fth* ^+/+^/*Ftl* ^+/-^ mice (29%). Mice with the *Fth* ^+/-^/*Ftl* ^-/-^ genotype were not observed.

### Systemic iron homeostasis in Ftl knock-out mice

**Fig 2:**
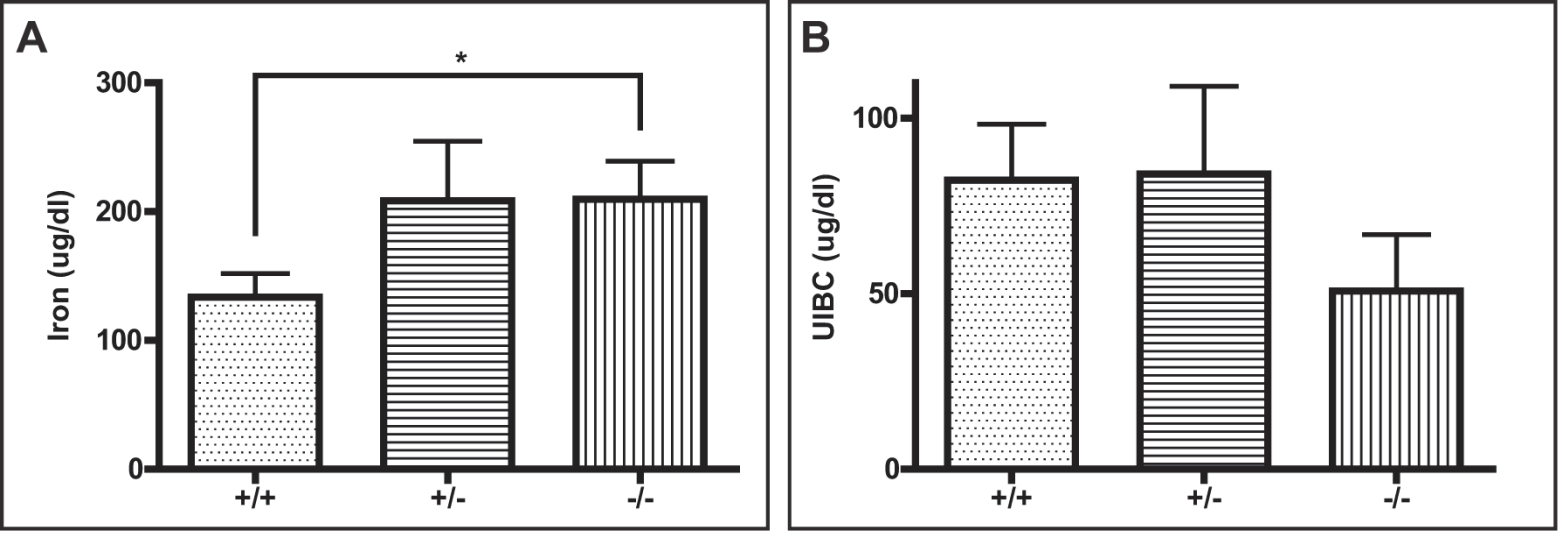
Deletion of one or both alleles of the *Ftl* gene causes a mild increase of serum iron levels that was significant for Ftl^-/-^ mice (*p* < 0.05) (A), and a decrease of UIBC levels in Ftl^-/-^ mice that did not reach statistical significance (*p* < 0.06) (B). Wild-type control (+/+; n = 12), *Ftl* ^+/-^ (+/-; n = 8), and *Ftl* ^-/-^ (-/-; n = 10) mice were analyzed. Samples were analyzed by a two-tailed *t*-test and results considered significant for *p* < 0.05.

**Fig 3:**
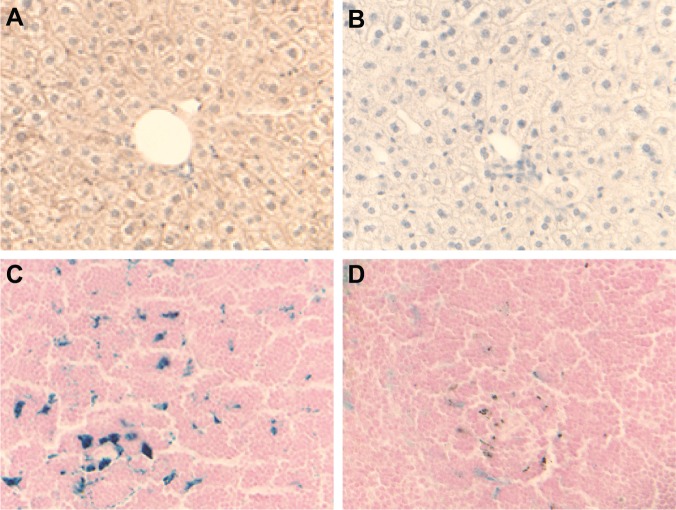
Histological and immunohistochemical studies on paraffin embedded sections from wild type and *Ftl* knock-out mice. Sections shown are from the liver immunostained with antibodies against the L chain (A, B) and from the spleen stained with the Perls’ Prussian blue method (C, D). Sections are from seven month-old wild-type mice (A, C) and from a twelve month-old *Ftl* ^-/-^ mouse (B) and a seven month-old *Ftl* ^-/-^ (D) mouse. Original magnification x40.

**Fig 4:**
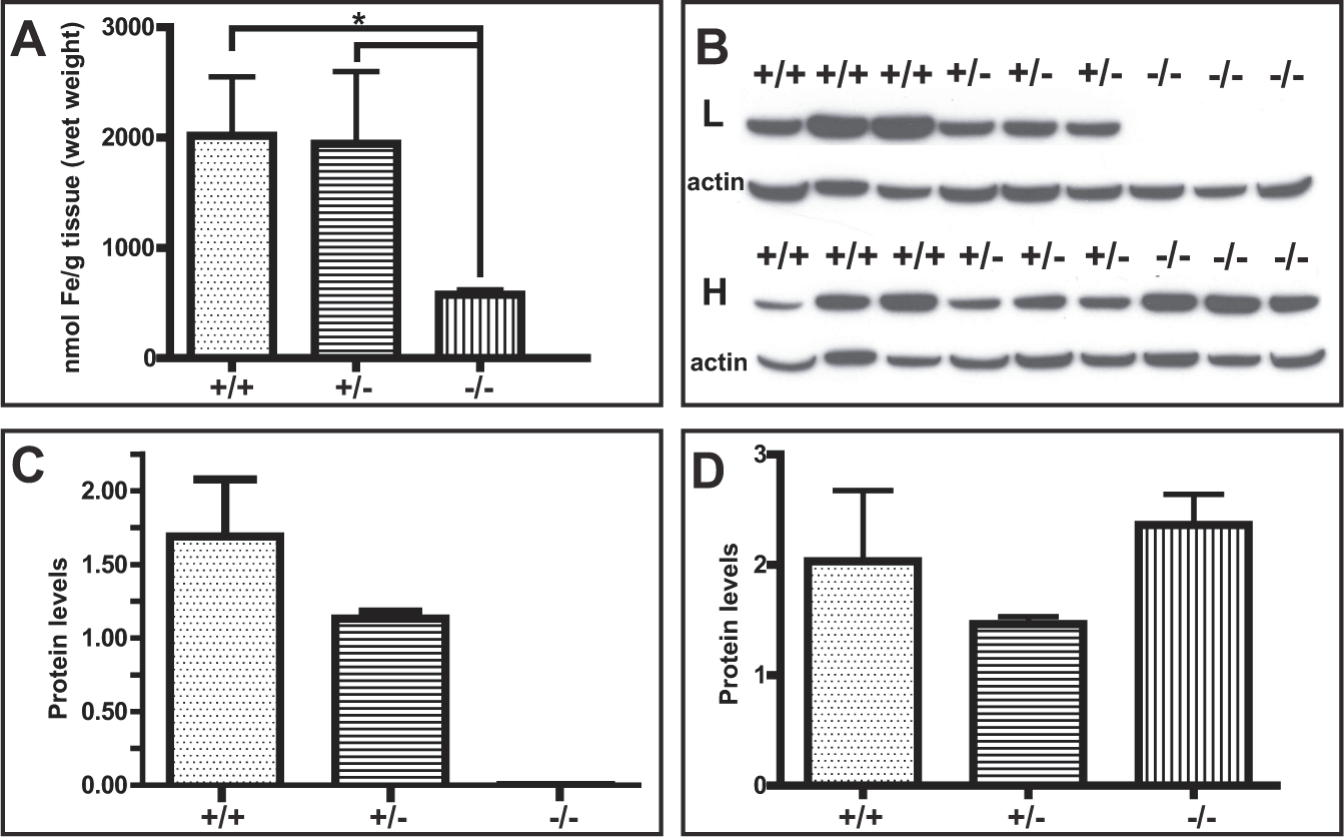
Measurement of total iron levels in the liver showed significant changes between Ftl^-/-^ knock-out mice (n = 4) and wild-type (+/+; n = 5) mice (*p* < 0.05) and Ftl^+/-^ heterozygous (+/-; n = 4) mice (*p* < 0.05). *Ftl* ^+/-^ values were not significantly different from those of wild-type mice (A). Western blot analysis of protein samples from the liver using antibodies specific for the L and H chains. Membranes were reblotted for β-actin to determine equal protein loading (B). Densitometric analysis from three independent experiments showed that L levels were decreased in *Ftl* ^+/-^ (+/-; n = 4) mice compared to age-matched wild-type mice (+/+; n = 4), although the change did not reach statistical significance. The knock-out of the *Ftl* gene (-/-; n = 4) led to the complete loss of immunoreactivity for the L subunit (B, C). Analysis of the H subunit in the same samples did not show any significant differences between wild-type, *Ftl* ^+/-^, and *Ftl* ^-/-^ mice (D).

To assess the impact of the knock-out of the *Ftl* gene on the major systemic iron utilization pathway, we determined serum iron levels, unsaturated iron binding capacity (UIBC), and hematological parameters in single (*Ftl* ^+/-^) and double (*Ftl* ^-/-^) *Ftl* knock-out mice. Compared to wild-type mice, an increase in serum iron levels was observed in both, *Ftl* ^+/-^ and *Ftl* ^-/-^ mice; however, this increase was significant (*p* < 0.05) only in *Ftl* ^-/-^ mice (Fig. 2A). Compared to wild-type mice, UIBC levels were decreased in *Ftl* ^-/-^ mice; however, this change did not reach statistical significance (*p* < 0.06) (Fig. 2B). No significant differences were observed on red cells and leukocyte counts, as well as on hematocrit and serum hemoglobin values. Significant elevation of the values of mean corpuscular hemoglobin (MCH), mean corpuscular hemoglobin concentration (MCHC), and red cell distribution width (RDW) was observed in *Ftl* ^-/-^ mice. Compared to wild-type controls, the mean corpuscular volume (MCV) values were also elevated in *Ftl* ^-/-^ mice, but did not reach statistical significance (Table 1). Pathologic analysis of *Ftl* ^-/-^ mice did not reveal any significant differences with wild-type control mice. No gross tissue abnormalities were observed in sections from the heart, muscle, liver, stomach, intestine, spleen, adipose tissue, lungs, and reproductive organs (testis and ovaries). Immunohistochemical analysis using antibodies against the L subunit revealed complete lack of immunoreactivity in sections from *Ftl* ^-/-^ mice (Fig. 3). Histochemical analysis showed diminished iron deposition in reticuloendothelial cells of *Ftl* ^-/-^ mice (Fig. 3C, D) compared to wild-type control mice. Although we were not able to detect iron deposition in the liver by Perls’ Prussian blue method, as previously reported for other mouse models [19], we observed a significant decrease in total iron content in the liver of *Ftl* ^-/-^ knock-out mice. A significant difference in total iron levels was also observed between *Ftl* ^+/-^ and *Ftl* ^-/-^ mice, with *Ftl* ^+/-^ values not significantly different from those of wild-type mice (Fig. 4A). Western blot analysis of protein samples from the liver showed that the levels of the L subunit were decreased in *Ftl* ^+/-^ mice compared to wild-type mice; however, this change did not reach statistical significance. Knock-out of the *Ftl* gene led to the complete loss of immunoreactivity for the L subunit (Fig. 4B, C). Analysis of the H subunit in the same samples did not show any significant differences between wild-type, *Ftl* ^+/-^, and *Ftl* ^-/-^ mice (Fig. 4B, D).

**Table 1:** Hematological parameters of *FTL* knock-out mice compared to wild-type mice (*Ftl* ^*+/+*^).

	RBC	WBC	Hb	HtC	MCV	MCH	MCHC	RDW
Ftl^+/+^	8.9 ± 0.3	5.4 ± 0.8	11.6 ± 0.4	38.1 ± 1.4	42.6 ± 0.7	12.9 ± 0.1	30.4 ± 0.3	17.6 ± 0.2
Ftl^+/-^	10.4 ± 0.6	7.1 ± 1.6	12.5 ± 0.4	44.3 ± 4.1	42.4 ± 1.6	12.2 ± 0.6	29.2 ± 1.8	20.9 ± 2.7
Ftl^-/-^	8.8 ± 0.5	5.6 ± 0.5	12.5 ± 0.7	38.8 ± 2.3	44.1 ± 1.1	13.9 ± 0.4*	31.4 ± 0.3*	18.7 ± 0.5*

We determined blood cell indices on 6- to 7-month-old wild type (*n =* 12), *Ftl* ^*+/-*^ (*n =* 7), and *Ftl* ^*-/-*^ (*n =* 11) animals. The following hematological parameters were measured: RBC, red blood cells number (x 10^6^/ml); WBC, white blood cells (x 10^3^/ml); Hb, hemoglobin (g/dl); HtC, hematocrit (%), mean corpuscular volume (MCV), mean corpuscular hemoglobin (MCH), mean corpuscular hemoglobin concentration (MCHC), and red cell distribution width (RDW). Significant differences compared to controls (p < 0.05) are indicated by *. Values are mean ± SEM.

### Iron homeostasis in the brain of Ftl knock-out mice

**Fig 5:**
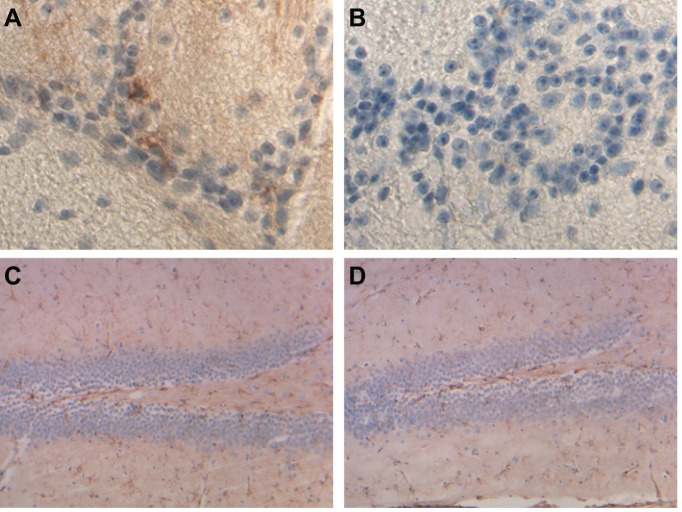
Immunohistochemical studies on paraffin embedded sections from wild type and *Ftl* knock-out mice. Sections shown are from the olfactory bulb immunostained with antibodies against the L chain (A, B) and from the hippocampus immunostained with antibodies against GFAP (C, D). No immunoreactivity is seen for the L subunit in knock-out mice (B). No significant differences were observed in GFAP staining between wild type and knock-out mice (C, D). Sections are from seven month-old wild-type (A, C) and *Ftl* ^-/-^ (B, D) mice. Original magnification x40.

**Fig 6:**
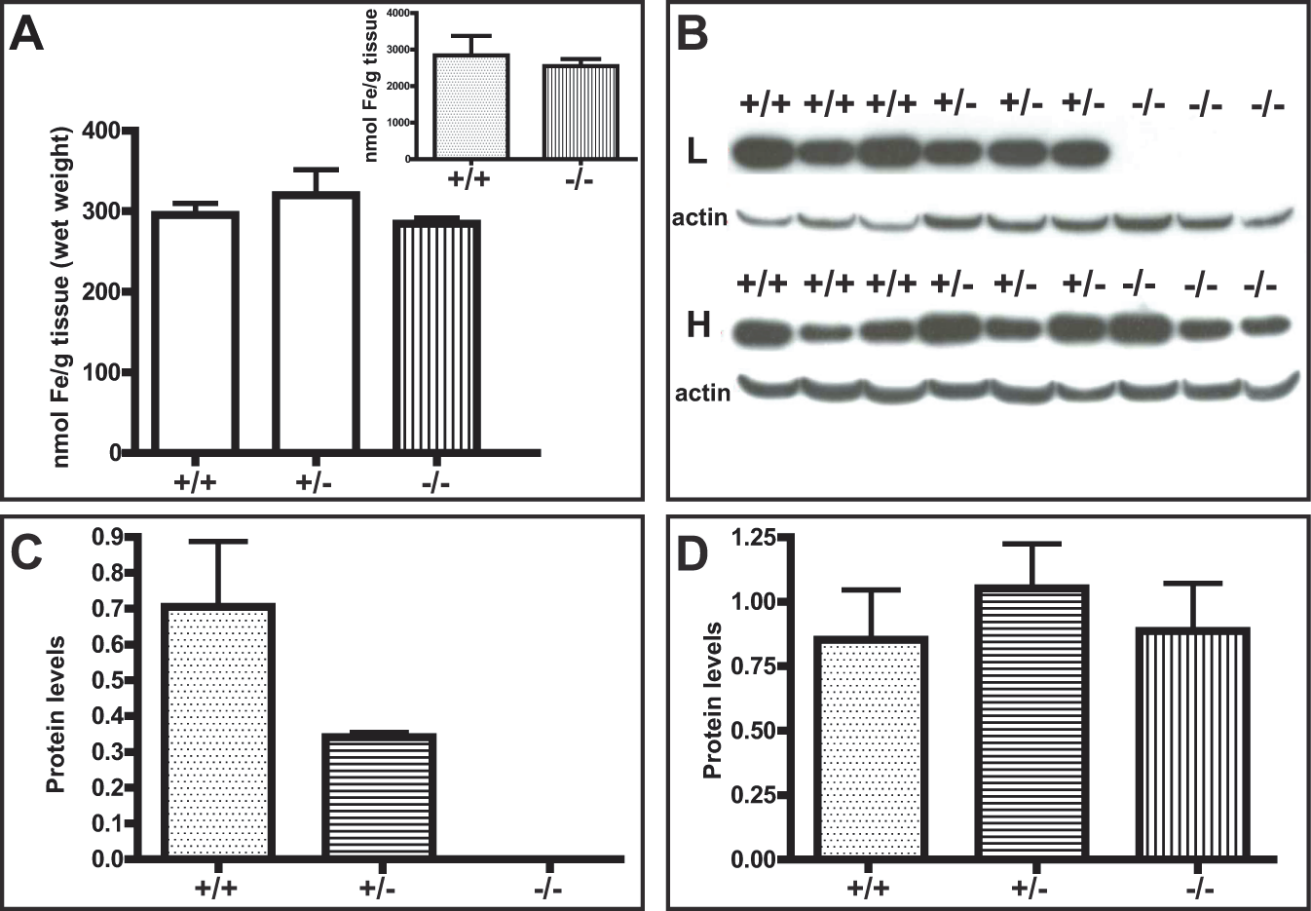
Measurement of total iron levels in the cerebral cortex did not show significant changes between wild-type mice (+/+; n = 5), Ftl^+/-^ heterozygous mice (+/-; n = 4), and Ftl^-/-^ knock-out mice (n = 4) (A). No significant changes were observed in the striatum between wild-type and *Ftl* knock-out mice (inset). Western blot analysis of protein samples from the cerebral cortex using antibodies specific for the L and H chains. Membranes were reblotted for β-actin to determine equal protein loading (B). Densitometric analysis from three independent experiments showed that L levels were decreased in *Ftl* ^+/-^ (+/-; n = 4) mice compared to age-matched wild-type mice (+/+; n = 4), although the change did not reach statistical significance. The knock-out of the *Ftl* gene (-/-; n = 4) led to the complete loss of immunoreactivity for the L subunit (B, C). Analysis of the H subunit in the same samples did not show any statistically significant differences between the three genotypes (D).

To assess the impact of the knock-out of the *Ftl* gene on brain iron metabolism, we compared brains of *Ftl* ^-/-^ and wild-type mice. No significant morphological differences were observed on H&E stainings. Abnormal iron deposition in *Ftl* ^-/-^ mice was not observed by Perls’ Prussian blue method. Immunohistochemical analysis using antibodies against the L subunit revealed complete lack of immunoreactivity in sections from *Ftl* ^-/-^ mice (Fig. 5A, B). Immunohistochemistry using antibodies against GFAP showed the presence of numerous GFAP-positive reactive astrocytes throughout all neocortical areas. This immunoreactivity was not different between wild-type controls and *Ftl* ^-/-^ mice (Fig. 5C, D). No significant changes in total iron content in the cerebral cortex (CTX) and striatum (caudate and putamen nuclei) were observed between wild-type and *Ftl* ^-/-^ knock-out mice (Fig. 6A). Western blot analysis showed a decrease in the levels of the L subunit in heterozygous *Ftl* ^+/-^ mice; however, this change did not reach statistical significance (Fig. 6B, C). Knock-out of the *Ftl* gene led to the complete loss of detection of the L subunit by western blot (Fig. 6B, C). Analysis of the same samples did not show significant differences in the levels of the H subunit between wild-type, *Ftl* ^+/-^, and *Ftl* ^-/-^ mice (Fig. 6B, D).

### Effects of the deletion of the ferritin genes on the expression of genes of iron metabolism

We analyzed by multiplex RT-PCR a total of 18 genes (genes and accession numbers are listed in S1 Table) that play a role in iron metabolism and related pathways. Analysis was performed in triplicate and expression was normalized to the *Polr2a* as previously described [14]. We analyzed liver and CTX tissue samples from wild-type, *Ftl* ^+/-^ heterozygous, *Ftl* ^-/-^ homozygous, *Fth* ^*+/-*^ heterozygous, and *Fth* ^+/-^/*Ftl* ^+/-^ double heterozygous mice. Expression levels were compared between mutant and wild-type mice. Analysis of *Ftl* ^+/-^ mice showed no significant differences in the expression of the genes analyzed in the liver. Lower levels of *Ftl mRNA* and higher levels of *hepcidin* (*Hamp) mRNA* were observed, but they did not reach statistical significance (S1 Fig.). In the CTX of *Ftl* ^+/-^ mice, a significant decrease in the expression of *Ftl* (*p*<0.0001) and *Trfrc* (*p*<0.01) genes was observed. A decrease in the levels of *Hmox1* was observed, but it did not reach statistical significance (S2 Fig.). Analysis of *Ftl* ^-/-^ mice showed no significant differences in the expression of the genes analyzed, except for the complete lack of expression of *Ftl* in both, liver and brain samples (S3 and S4 Figs.). Lower levels of liver *Trfrc mRNA* and brain *Hmox1* were observed, but the changes in values did not reach statistical significance (S3 and S4 Figs.). In summary, no significant changes were observed in the expression levels of the majority of the genes analyzed, with both heterozygous and homozygous knock-out mice showing similar trends. Analysis of liver gene expression in *Fth* ^+/-^/*Ftl* ^+/-^ double heterozygous mice did not reveal significant differences with wild-type mice; however, analysis of gene expression in the brain showed significant changes in a number of genes (S5 and S6 Figs.). A significant decreased in gene expression values were observed for *AcoI* (*p*<0.02), *Fxn* (*p*<0.002), *Dmt1* (*p*<0.0005), *Fth1* (*p*<0.0003), *Ftl* (*p*<0.0007), *Sod1* (*p*<0.01), *Sod2* (*p*<0.01), and *Trfrc* (*p*<0.006) (S6 Fig.). Gene expression analysis in *Fth* ^*+/-*^ mice was done with mRNA isolated from the CTX. Significant changes were observed for Cp (*p*<0.02), *AcoI* (*p*<0.02), *Dmt1* (*p*<0.003), Abcb7 (*p*<0.01), *Fth1* (*p*<0.0001), *Hmox1* (*p*<0.0003), *Tfrc* (*p*<0.0001), *Tf* (*p*<0.0001), *Ireb2* (*p*<0.0001), and *Pank2* (*p*<0.02) (S7 Fig.).

## Discussion

We generated mice in which the murine ferritin genes were disrupted by homologous recombination to evaluate the biological significance of loss of iron storage function in mammalian iron homeostasis. Homozygous loss of the *Ftl* allele on a wild-type *Fth* background caused embryonic lethality in approximately 50% of the expected *Ftl* ^-/-^ embryos. Surviving animals were not phenotypically different from wild-type control mice, with animals showing some abnormal neurological features as they grew older (head tilt and a circling behavior in the same direction), which persisted throughout the life of the animal. Spinning or head tilt usually indicate damage to the vestibular system, which has been attributed to different factors, including otitis media, arteritis, and central nervous system lesions. Since the phenotype was not observed in wild-type littermates, a bacterial infection was not expected to be the cause. Further studies may help clarify the origin and pathogenic mechanisms involved in the development of the neurologic phenotype seen in *Ftl* ^-/-^ mice. Although both ferritin subunits have different functions and the ratio of the two subunits in the complex depends on the relative expression levels of the two genes, complete knock-out of the *Ftl* allele reveals that mice with H ferritin homopolymers have the capacity to sequester iron without substantial changes in iron homeostasis *in vivo*. We also observed that complete knock-out of *Fth* causes embryonic lethality that cannot be rescued by the L subunit alone, as previously reported [17, 18]. Thus, our data support the concept that the H subunit plays an important role during embryogenesis, since the L subunit is not able to rescue the loss of the H subunit. Importantly, just like the H subunit, the L subunit may have an important role in mouse embryogenesis since the H subunit cannot rescue the complete loss of the L subunit in 100% of the cases. Breeding of mice with disrupted *Fth* and *Ftl* alleles revealed that some degree of complementation between the subunits must occur since we observed complete embryonic lethality when only one *Fth* allele was present (*Fth* ^+/-^) in the *Ftl* ^*-/-*^ knock-out background (*Fth* ^+/-^/*Ftl* ^*-/-*^ mice). These data suggest that during embryogenesis, a minimal level of ferritin expression must be achieved for survival; with at least two ferritin subunits present, one of which being an H subunit.

The phenotype of *Ftl* ^*-/-*^ knock-out mice was assessed by measuring parameters of iron status that were compared with those of wild-type controls. Homozygous loss of *Ftl* did not lead to significant changes in Hb, Hct, and RBC values, but *Ftl* ^-/-^ mice had the hematological phenotype of macrocytic red cells with significant increase in MCH, MCHC, and RDW values, and an elevated MCV value that did not reach statistical significance. Macrocytic red cells can be produced due to a number of factors, including low levels of vitamin B12. An increase in serum iron (Fe (III) bound to serum transferrin) was also observed in *Ftl* ^-/-^ mice. Since the mice did not have L subunits, we did not attempt to measure blood levels of L subunits to assess body iron storage. Compared to wild-type controls, we observed reduced ferric iron deposition in the spleen of *Ftl* ^-/-^ mice, suggesting some problem in iron retention. Since we were not able to detect iron in the liver by Perls’ Prussian blue method, iron levels in the liver were assessed by measuring total iron content [19, 20]. Interestingly, we observed a significant decrease in total iron content in the liver of *Ftl* ^-/-^ knock-out mice, which did not lead to significant changes at the transcriptional level, with heterozygous and homozygous *Ftl* ^-/-^ knock-out mice showing similar mRNA profiles. Western blot analysis demonstrated the complete loss of the L subunit in *Ftl* ^-/-^ knock-out mice, without over production of the H subunit in the liver. Thus, inactivation of the *Ftl* allele did not cause up-regulation of the *Fth* allele or significant changes at the transcriptional level in the liver, but some problem in body iron storage was noted. Analysis of gene expression in the liver of *Fth* ^+/-^/*Ftl* ^+/-^ double heterozygous knock-out mice showed a liver gene expression pattern similar to the one observed for the single heterozygous knock-out mice, without significant differences with wild-type mice. No significant differences were observed between males and females *Ftl* ^-/-^ knock-out mice.

Iron is a metal that is required as a cofactor in many metabolic processes in the CNS, including oxidative phosphorylation, neurotransmitter production, nitric oxide metabolism, and oxygen transport [2, 21]. However, diseases associated with loss of function of proteins involved in iron metabolism rarely result in either brain iron overload or deficiency or neurologic disease. Recently, an individual homozygous for a nonsense mutation in exon 3 of the *FTL* gene has been reported [12]. In this patient, no L subunit could be found. The patient had seizures during infancy and presented with an atypical form of RLS, iron deficiency with mild neuropsychological impairment, a reduced intelligence quotient and important progressive hair loss; however, erythropoiesis and systemic iron homeostasis did not seem to be affected. Analysis of fibroblasts and induced neurons derived from the patient showed alterations of cellular iron homeostasis and oxidative stress. Interestingly, no signs of iron accumulation in the basal ganglia was detected by MRI. *Ftl* ^-/-^ mice seem to recapitulate several aspects of this condition, including signs of iron deficiency (diminished iron deposition in reticuloendothelial cells and significant low iron levels in the liver), with normal blood cells counts, hematocrit and serum hemoglobin values, and without significant changes in brain iron metabolism. Further studies, including behavioral and cellular studies using cell lines derived from *Ftl* ^-/-^ mice will establish whether *Ftl* ^-/-^ mice could be used to model different aspects of this condition and to test some form of therapeutic intervention.

A direct genetic link between abnormal iron metabolism and neurodegeneration has been established in the neurodegenerative diseases Aceruloplasminemia, an autosomal recessive disorder caused by mutations in the *ceruloplasmin* gene, and HF, an autosomal dominant disorder caused by mutations in the *FTL* gene [1, 2, 10, 11, 21]. In HF, two key pathogenic mechanisms have been proposed for the development of the disease: a loss of normal ferritin function (with decreased iron incorporation) that triggers intracellular iron accumulation and overproduction of ferritin polypeptides, and a gain of toxic function through radical production, ferritin aggregation, and oxidative stress [1, 2]. Since the disease is caused by mutations in the *FTL* gene [1], it was important to determine whether loss of function of the L subunit in the brain could lead to some of the pathologic features observed in HF. The brains of *Ftl* ^-/-^ mice did not show signs of neurodegeneration, presence of an inflammatory process, noticeable protein aggregates, or iron accumulation as in patients with HF and an animal model for the disease [13]. Our data suggest that the deleterious effect(s) caused by mutant L subunits in HF are driven by disruption of the ferritin pore structure and unraveling of the C-terminus of mutated L subunits in the heteropolymer rather than by a loss of normal function of the L subunit itself [8]. Importantly, our data is very significant for the development of potential treatment for HF using RNA interference to induce sequence-specific post-transcriptional gene silencing of mutant *FTL*. Since H-ferritin homopolymers are capable of maintaining brain iron homeostasis, RNA interference could be used as a therapeutic approach even if the inhibition of the expression of the mutant allele suppresses in part (or to a high degree) the expression of the wild-type *FTL* allele.

As previously reported [17, 18] and as found in this work, complete knock-out of the *Fth* gene in mice on a wild-type *Ftl* background causes embryonic lethality. The deletion of one allele of the *Fth* gene in the presence of wild-type *Ftl* is not embryonic lethal and these mice are not significantly different from normal mice. Analysis of the expression of several iron-related genes in the brain of a previously reported *Fth* heterozygous knock-out model [18] showed a decrease in the expression of *Fth* and *Tfrc* as we observed here. The authors also noted no changes in the expression of *Tf*, *Ftl*, *Dmt1*, and *Cp*. As reported by Ferreira and collaborators [22] we did not observe significant changes in *Ftl* expression levels. In addition, we observed a significant reduction in gene expression for *Tf*, *Dmt1*, and *Cp*, compared to wild type control mice. We also noticed changes in the expression of additional genes involved in iron metabolism. These differences may be due to the use of different brain areas and methodologies for the different studies. Interestingly, the same authors [22] reported normal total brain iron with a protein profile of iron deficiency determined by western blot analysis. In our case, the profile generated by the multiplex study suggests a profile more associated with increased cerebral iron since we observed decreased in the levels of *Tf* and *Trfrc*, while the lower levels of *Fth* mRNA were most likely the result of a gene dose effect. Moreover, we observed a significant decrease in *Cp* levels, which has been associated with increased iron accumulation and free radical injury in the CNS of *Cp* ^-/-^ mice [23]. When only one functional *Ftl* allele (*Ftl* ^*+/-*^) was present in the heterozygous *Fth* ^+/-^ background (*Fth* ^+/-^/*Ftl* ^+/-^ mice), we observed a significant decrease in the expression of *AcoI*, *Fxn*, *Dmt1*, *Fth*, *Ftl*, *Sod1*, *Sod2*, and *Trfrc* genes, similar to the gene expression patterns observed for *Fth* ^+/-^ and *Ftl* ^+/-^ single heterozygous knock-out mice in the brain. Interestingly, *Fth* ^+/-^/*Ftl* ^+/-^ double heterozygous knock-out mice showed a significant decrease in the expression of *Sod1* and *Sod2*, the enzymes responsible for destroying free superoxide radicals in the cytoplasm and mitochondria, respectively. A decrease in SOD activity was reported previously in the single heterozygous *Fth* ^+/-^ knock-out mice [18], although no changes in gene expression were detected in the model as well as in our single *Fth* ^+/-^ knock-out mouse model. Further work may clarify the mechanism(s) associated with the lower levels of expression of *Sod1* and *Sod2* in the *Fth* ^+/-^/*Ftl* ^+/-^ double heterozygous model, and whether the decrease in SOD activity observed in heterozygous *Fth* ^+/-^ knock-out mice is associate with small changes in the expression of the *Sod* genes. In *Fth* ^+/-^/*Ftl* ^+/-^ double heterozygous mice, the decrease in the expression of the *Sod* genes may lead to a more significant decrease in SOD activity, making them more susceptible to oxidative stress damage compared to single heterozygous knock-out mice.

The generation of mouse models with disrupted ferritin alleles provides novel tools in which to study the specific role of each ferritin subunit *in vivo*. Moreover, the study of these models will lead a better understanding of the interaction between both ferritin subunits during embryonic development and systemic iron homeostasis *in vivo*. In addition, they may provide important clues of the role of iron dyshomeostasis in the brain in neurodegenerative diseases.

## Supporting Information

S1 Fig Multiplex RT-PCR expression analysis of iron metabolism related genes in the liver. Bar graphs depict differential gene expression levels between 7 month old Ftl^+/-^ (+/-) and wild-type age-matched control mice (+/+). Analysis was performed in triplicate and normalized to the *Polymerase II polypeptide A* gene (*Polr2a*). The group averages are reported as relative mRNA levels mean ± SD. Differences in gene expression were determined by two-tailed *t*-test.
(EPS)

S2 Fig Multiplex RT-PCR expression analysis of iron metabolism related genes in the cerebral cortex. Bar graphs depict differential gene expression levels between 7 month old Ftl^+/-^ (+/-) and wild-type age-matched control mice (+/+). Analysis was performed in triplicate and normalized to the *Polr2a* gene. The group averages are reported as relative mRNA levels mean ± SD. Differences in gene expression were determined by two-tailed *t*-test.
(EPS)

S3 Fig Multiplex RT-PCR expression analysis of iron metabolism related genes in the liver. Bar graphs depict differential gene expression levels between 7 month old Ftl^-/-^ knock-out mice (-/-) and wild-type age-matched control mice (+/+). No expression was detected for the L subunit. Analysis was performed in triplicate and normalized to the *Polr2a* gene. The group averages are reported as relative mRNA levels mean ± SD. Differences in gene expression were determined by two-tailed *t*-test.
(EPS)

S4 Fig Multiplex RT-PCR expression analysis of iron metabolism related genes in the cerebral cortex. Bar graphs depict differential gene expression levels between 7 month old Ftl^-/-^ knock-out mice (-/-) and wild-type age-matched control mice (+/+). No expression was detected for the L subunit. Analysis was performed in triplicate and normalized to the *Polr2a* gene. The group averages are reported as relative mRNA levels mean ± SD. Differences in gene expression were determined by two-tailed *t*-test.
(EPS)

S5 Fig Multiplex RT-PCR expression analysis of iron metabolism related genes in the liver. Bar graphs depict differential gene expression levels between 7 month old Fth^+/-^/Ftl^+/-^ double heterozygous mice (het/het) and wild-type age-matched control mice (+/+). Analysis was performed in triplicate and normalized to the *Polr2a* gene. The group averages are reported as relative mRNA levels mean ± SD. Differences in gene expression were determined by two-tailed *t*-test.
(EPS)

S6 Fig Multiplex RT-PCR expression analysis of iron metabolism related genes in the cerebral cortex. Bar graphs depict differential gene expression levels between 7 month old Fth^+/-^/Ftl^+/-^ double heterozygous mice (het/het) and wild-type age-matched control mice (+/+). Analysis was performed in triplicate and normalized to the *Polr2a* gene. The group averages are reported as relative mRNA levels mean ± SD. Differences in gene expression were determined by two-tailed *t*-test.
(EPS)

S7 Fig Multiplex RT-PCR expression analysis of iron metabolism related genes in the cerebral cortex. Bar graphs depict differential gene expression levels between 4 month old Fth^+/-^ heterozygous mice (+/-) and wild-type age-matched control mice (+/+). Analysis was performed in triplicate and normalized to the *Polr2a* gene. The group averages are reported as relative mRNA levels mean ± SD. Differences in gene expression were determined by two-tailed *t*-test.
(EPS)

S1 Table Genes and Primers (without universal tags) used in Multiplex RT-PCR Gene Expression Analysis. (DOCX)
