# Supplementary material for: Systemic and Cerebral Iron Homeostasis in Ferritin Knock-Out Mice
Source: PLoS One. 2015 Jan 28;10(1):e0117435. doi: 10.1371/journal.pone.0117435 (PMC4309591; doi:10.1371/journal.pone.0117435)
Supplement: S1 Table — (DOCX) [file pone.0117435.s008.docx]

**RefSeq Gene Symbol Forward Primer Reverse Primer**

NM_009592 Abcb7 tgcagttacacggtggagaa caaaattcagcattgccaga

NM_007393 Actb atacaccatgtacccaggcatt agggtgtaaaacgcagctca

NM_001042611 Cp tttacccagcaacgctctct tctttatgtggccttttggg

NM_008732 Dmt1 ctgatcgtctgctccatcaa cccaatgcaatcaaacactg

NM_010239 Fth1 ctcatgaggagagggagcat gtgcacactccattgcattc

NM_010240 Ftl1 gtcccgtggatctgtgtct aggagctaaccgcgaagaga

NM_008044 Fxn tggtgcatttgaggaacttg tcctccagggtatagggctt

NM_008084 Gapdh atcactgccacccagaagac agatccacgacggacacatt

NM_032541 Hamp catgatggcactcagcactc ggtcaggatgtggctctagg

NM_010442 Hmox1 ttaccttcccgaacatcgac ctagcaggcctctgacgaag

NM_010443 Hmox2 gtagggcaaaatgagccaga caaattcaggtccaaggcat

NM_007386 Irp1 gaggcctaacaccacgagag agtctgaggtgcctgcttgt

NM_022655 Irp2 tgaagaaacggacctgctct gctcacatccaaccacctct

NM_153501 Pank2 cttacggatccacaggcatt ccagttttcgaagctgaagg

NM_027153 Pir cgaaggtttacactcgcaca aaaatggcttcttttggggt

NM_009089 Polr2a tcgagacaaaactggctcct gaggaagagtccgatgcttg

NM_007475 Rplp0 cttcattgtgggagcagaca gaggtcctccttggtgaaca

NM_011434 Sod1 ggggacaatacacaaggctgt agtcacattgcccaggtctc

NM_013671 Sod2 ctgtgggagtccaaggttc agcggaataaggcctgttgt

NM_011638 TfR-1 ttgggtagttggagattgcc tgaggtctttggcttctggt

NM_133977 Trf ataccgatgctatgaccttggat caggacttcttgccttcgag

NM_015799 Trfr2 ccatcagtgctgacattgct tgggggtagagactctgtgg

**Supplemental Table 1**
